# Circular RNA and intervertebral disc degeneration: unravelling mechanisms and implications

**DOI:** 10.3389/fmolb.2023.1302017

**Published:** 2023-12-19

**Authors:** Guohao Xie, Tingrui Wu, Guangju Ji, Hang Wu, Yue Lai, Bo Wei, Wenhua Huang

**Affiliations:** ^1^ Orthopaedic Center, Affiliated Hospital of Guangdong Medical University, Zhanjiang, China; ^2^ Guangdong Engineering Research Center for Translation of Medical 3D Printing Application, Guangdong Provincial Key Laboratory of Medical Biomechanics, National Key Discipline of Human Anatomy, School of Basic Medical Sciences, Southern Medical University, Guangzhou, China; ^3^ Guangdong Medical Innovation Platform for Translation of 3D Printing Application, The Third Affiliated Hospital of Southern Medical University, Guangzhou, China

**Keywords:** lower back pain, intervertebral disc degeneration, nucleus pulposus cell, extracellular matrix, circular RNA (circRNA)

## Abstract

Low back pain (LBP) is a major public health problem worldwide and a significant health and economic burden. Intervertebral disc degeneration (IDD) is the reason for LBP. However, we have not identified effective therapeutic strategies to address this challenge. With accumulating knowledge on the role of circular RNAs in the pathogenesis of IDD, we realised that circular RNAs (circRNAs) may have tremendous therapeutic potential and clinical application prospects in this field. This review presents an overview of the current understanding of characteristics, classification, biogenesis, and function of circRNAs and summarises the protective and detrimental circRNAs involved in the intervertebral disc that have been studied thus far. This review is aimed to help researchers better understand the regulatory role of circRNAs in the progression of IDD, reveal their clinical therapeutic potential, and provide a theoretical basis for the prevention and targeted treatment of IDD.

## 1 Introduction

Low back pain (LBP) is a major public health concern worldwide, and up to 84% of people experiences LBP during their lifetime. Approximately 23% of people experiences chronic LBP, and 11%–12% of people experiences LBP-related disability (Hoy et al., 2012). LBP affects people of all ages and is a major contributor to the global disease burden. For academics and physicians, managing LBP remains difficult despite advancements in its evaluation and treatment (Vlaeyen et al., 2018). A strong correlation exists between IDD and LBP (Risbud and Shapiro, 2014). According to modern evidence-based medicine, many factors cause or accelerate IDD development, including mechanical, genetic, traumatic, inflammatory, and biological factors (Molinos et al., 2015b; Oichi et al., 2020). There is a strong possibility that among these factors, genetic factors may cause the degeneration of the intervertebral disc (IVD) (Zhang et al., 2008).

The IVD is a complex fibrocartilaginous structure comprising the nucleus pulposus (NP), annulus fibrosus (AF), and cartilaginous vertebral endplates (CEP) (Liuke et al., 2005). The NP is a gelatinous structure located at the centre of the disc that produces the extracellular matrix (ECM), including aggrecan and type II collagen, which play crucial roles in maintaining the integrity of the disc (Roughley, 2004; Waxenbaum et al., 2021). The AF is a fibrous, avascular external region of the IVD consisting of type I and type II collagen fibrils that surround the NP (Ashinsky et al., 2019). As the main nutrient supply of the IVD is diffused by the endplates, a thin horizontal layer is necessary for the appropriate functioning of the IVD (Urban et al., 2004; Raj, 2008). Therefore, they play a crucial role in maintaining the proper function of the IVD. The development of IDD is associated with disorders or abnormalities in these three components.

The IVD is unique in that it does not have a rich blood and nutrient supply structure and is the largest non-vascular part of the body (Urban et al., 2004). The IVD cells depend on the capillary supply from the vertebral body (Roberts et al., 2006), and this unique structure of the IVD makes it highly vulnerable to injury, particularly in terms of the supply of nutrients to the disc. The IVD is very sensitive to glucose and oxygen, and a lack of nutrient supply reduces glucose concentration and increases lactate synthesis owing to a decrease in oxygen concentration, resulting in an acidic pH environment (Johnson et al., 2008; Hiyama et al., 2012). Cells have trouble surviving extended exposure to low pH or glucose concentrations because the rate of matrix formation substantially declines at an acidic pH and low oxygen concentrations, leading to disc degeneration (Horner and Urban, 2001; Urban and Roberts, 2003).

The main factors involved in IDD are apoptosis, ECM degradation, and inflammatory factors (Clouet et al., 2009). Metabolic disorders of NP cells under various factors reduce their ability to synthesise ECM components, such as aggrecan and type II collagen, and cause the secretion of a large number of ECM-degrading molecules, such as matrix metalloproteinases (MMP), deintegrins, and a disintegrin and metalloproteinase with thrombospondin-like motifs (ADAMTS), resulting in progressive degradation of the ECM, which is responsible for the mechanical functioning of the disc (Vo et al., 2013; Wang et al., 2015). The association between disc degeneration and inflammation is widely recognised. Inflammation also plays a significant role in disc degeneration, and inflammatory variables may even play a role in the pain induced by IDD (Richardson et al., 2009; Molinos et al., 2015a; Mosley et al., 2017).

**FIGURE 1:**
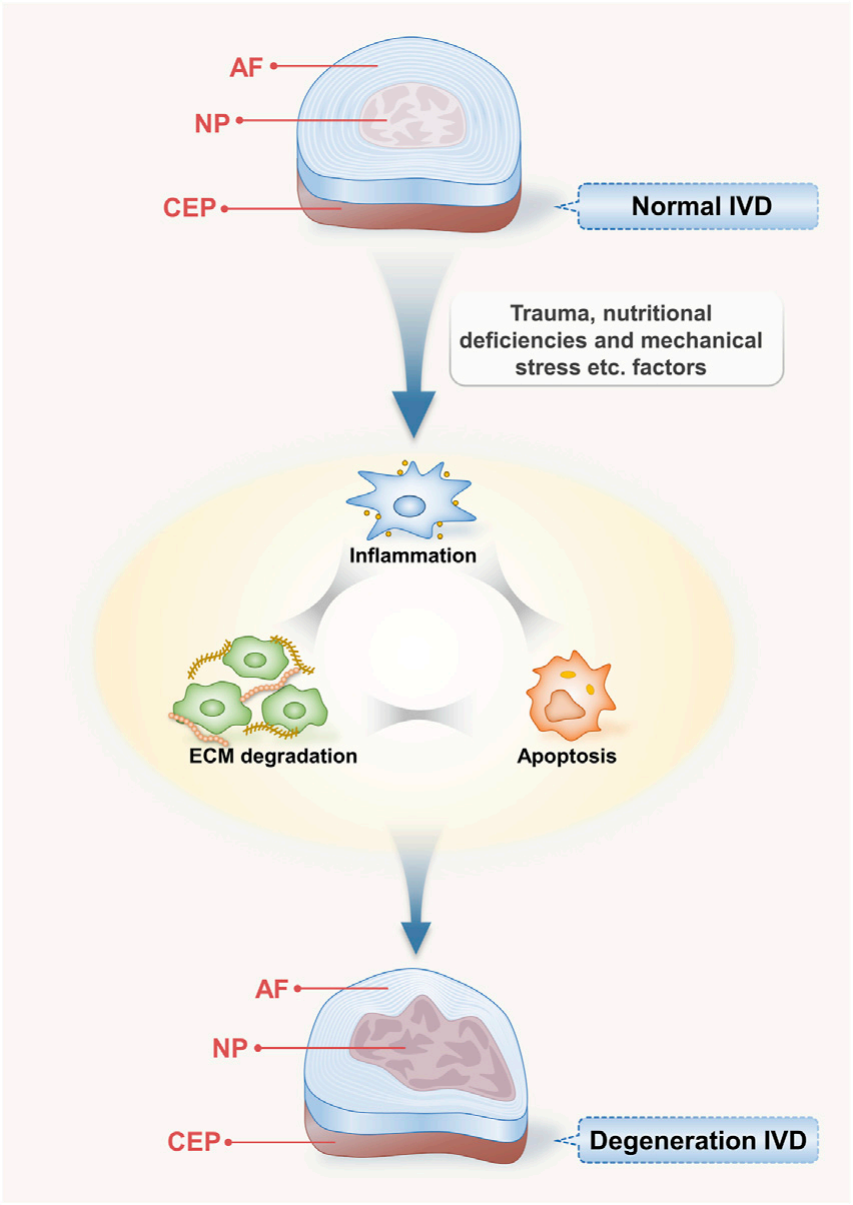
Structure of intervertebral disc and its degenerative process. The IVD is an intricate fibrocartilaginous formation comprising the nucleus pulposus (NP), annulus fibrosus (AF), and cartilaginous endplates (CEP); they play a crucial role in maintaining the proper function of the IVD. Various induction factors that affect the disc, such as trauma, malnutrition, and mechanical stress, can lead to inflammation, apoptosis, and ECM degradation, resulting in IDD.

NP cells secrete proinflammatory cytokines in response to multiple triggers, including tumour necrosis factor (TNF), interleukin-1β (IL-1β), and interleukin-6 (IL-6) (Le Maitre et al., 2005; Wang et al., 2011). Proinflammatory cytokines lead to the dysregulation of ECM metabolism by upregulating ECM degradation enzymes (e.g., MMP) and downregulating ECM structural components (e.g., type II collagen and aggrecan) (Séguin et al., 2005; Studer et al., 2011; Risbud and Shapiro, 2014). ECM degradation further stimulates NP cells to produce proinflammatory factors, creating a vicious cycle (Quero et al., 2013). Simultaneously, the senescence and death of NP cells are facilitated by metabolic abnormalities in the ECM and the generation of inflammatory substances (Wang et al., 2016). Apoptosis, the degradation of the ECM, and the production of inflammatory factors are interrelated and mutually reinforcing and together contribute to the degeneration of IVDs (Figure 1).

Currently, the main treatment options for disc degeneration include physiotherapy, medication, and surgery, depending on the symptoms and degree of disc degeneration (Knezevic et al., 2017). However, treatment methods and their effects are unsatisfactory. According to some statistics, 10%–40% of patients experiences ‘failed back surgery syndrome’ following surgery, a condition in which patients experience persistent chronic back pain after surgery (Thomson and Jacques, 2009). Patients frequently experience back pain, preventing them from carrying out their daily work and activities or living normally (French et al., 2018). There is an increased risk of developing an addiction to narcotics owing to the necessity for pain relief (Chen et al., 2019). Based on these findings, there is a pressing need to explore new, practical, and improved prognostic approaches to address disc degeneration. In this context, gene therapy based on a class of non-coding RNA biological factors, such as circRNAs, has attracted attention.

The role of non-coding RNAs in various diseases has attracted increasing attention, and the differential expression of circRNAs between patients with IDD and healthy controls has been increasingly investigated (Wang et al., 2019b; Guo et al., 2020a). CircRNAs also promote or inhibit disc degeneration by modulating several pathological processes, including proliferation, apoptosis, phagocytosis, and senescence in the NP and chondrocytes and the expression of key components of the ECM, anabolic regulators of the ECM such as MMP and ADAMTS, and inflammatory cytokines such as IL-1. However, the mechanism of action of circRNAs in IDD is not well understood, and further research is necessary.

## 2 Properties, classification, and functions of circRNAs

CircRNAs are produced by back-splicing linear precursor mRNAs (pre-mRNAs), a class of non-coding RNAs that are cyclised and covalently bonded (Ashwal-Fluss et al., 2014; Zimmerman et al., 2020). CircRNA molecules were first discovered in RNA viruses in 1976 and in eukaryotic cells in 1991 (Wang et al., 2019a). At that time, circRNAs were considered to be formed by mis-splicing (Cocquerelle et al., 1993). However, in subsequent in-depth studies, numerous circRNAs exhibited tissue- and disease-specific expression patterns with important biological functions (Rybak-Wolf et al., 2015; Kristensen et al., 2018; Nicolet et al., 2018), indicating the need for more research on circRNAs.

Currently, circRNAs are classified into three categories: exonic circular RNAs (ecRNAs), which account for approximately 85% of all circRNAs; exonic, intronic circular RNAs (eicRNAs); and circular intronic RNAs (ciRNAs), all of which are classified based on various exon–intron combinations in the final sequence (Di Agostino et al., 2020; Chen et al., 2021). RNAs include cyclic introns from pre-mRNAs and tRNA intron cyclic RNA (Schmidt et al., 2019). EcRNAs are abundant in the cytoplasm, whereas EIcRNAs are abundant in the nucleus (Li et al., 2015a). Exon-containing circRNAs are the final splicing products and are currently the main products of genome discovery and study (Hsiao et al., 2017). Currently, it is believed that the circular conformation of most circRNAs is formed through back-splicing of exons in precursor mRNA. During this process, the 5′-splicing site joins the upstream 3′- splice site to form a circRNA that is covalently linked at the end by a 3′–5′ phosphodiester bond. This is in contrast to canonical splicing, where the upstream 5′splice site is sequentially jointed to the downstream 3′splice site to produce linear RNA (Zhang et al., 2016; Pagliarini et al., 2020).

**FIGURE 2:**
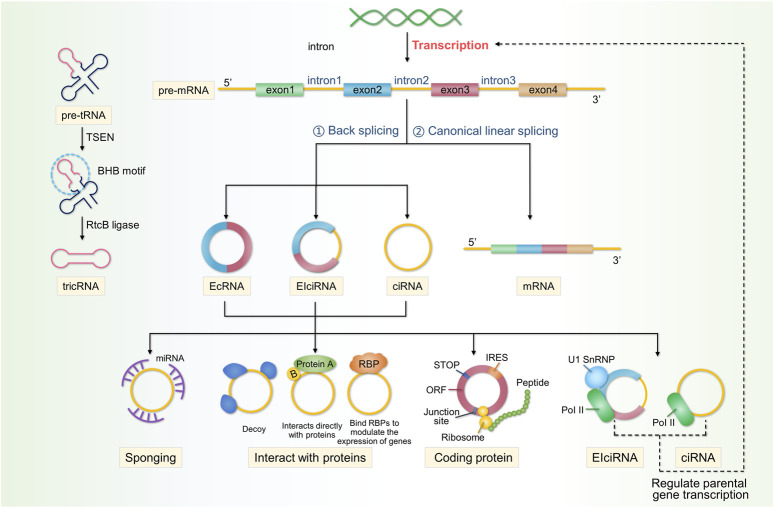
Properties, classification, and functions of circRNAs. CircRNAs are produced by the reverse splicing of linear precursor mRNAs (pre-mRNAs), followed by cyclisation. circRNAs are classified into three categories: EcRNAs, EIcRNAs, and ciRNAs. A small proportion of the ciRNAs formed from tRNA, known as tricRNA, which is formed by the TSEN complex cleaving the tRNA precursor at the canonical BHB motif to produce introns, is then joined together by RtcB ligase at the ends of the resulting introns. CircRNAs perform a multitude of functions within cells and can act as sponges for miRNAs. circRNAs interact with proteins in various ways and can affect the expression of parental genes. circRNAs derived from exonic RNA can be translated into encoded proteins. Additionally, circRNAs can compete with linear RNA to affect gene expression.

A small proportion of the circRNAs formed from tRNA, known as tricRNA, is a unique circular RNA formed by the tRNA splice endonuclease (TSEN) complex (consisting of two structural and two catalytic components), which cleaves the tRNA precursor at the canonical bulge-helix-bulge (BHB) motif to produce introns that are then joined together by RtcB ligase at the ends of the resulting introns (Paushkin et al., 2004; Schmidt et al., 2019). Back-splicing is a critical step in the creation of circRNAs and is controlled by trans-acting factors and cis-acting components (Chuang et al., 2018). Similar to that of other RNA transcripts, the abundance of circRNAs is controlled by their balanced relative rates of synthesis and degradation (Venkatesh et al., 2016). However, circRNAs are more stable than linear RNAs. Their half-life is at least 2-fold longer than that of linear RNAs, exceeding 48 h (Jeck et al., 2013; Enuka et al., 2016), and for some genes, they are 10-fold more abundant than the associated linear mRNAs (Borchardt et al., 2017). Moreover, circRNA expression is tissue- or cell-specific, suggesting the potential to be used as biomarkers for certain human diseases (Rybak-Wolf et al., 2015; Yang et al., 2017a). To date, the following circRNAs have been identified: sponging miRNAs, those interacting with proteins, those regulating parental gene expression, and those competing with linear RNAs (Li et al., 2020). Figure 2 shows the biogenesis and functions of circRNAs.

### 2.1 microRNA (miRNA) sponges

The role of circRNAs as a sponge has been the most extensively studied and is their main function. Most circRNAs are primarily located in the cytoplasm of the cell, which may be related to post-transcriptional regulation (Salzman et al., 2012; Yang et al., 2017c). MiRNAs are short RNA molecules, measuring of 19–25 nucleotides in length, that regulate the post-transcriptional silencing of target genes (Lu and Rothenberg, 2018). They regulate RNA stability and translation rates by pairing with complementary sites in target RNAs, called miRNA response elements (MREs) (Guo et al., 2010). According to the competitive endogenous RNA (ceRNA) theory, all RNA transcripts with miRNA-binding sites might interact and regulate one another by competing for the same miRNAs (Liu et al., 2019). Therefore, as long as circRNAs possess MREs that bind miRNAs, they can also act as endogenous RNAs that bind to miRNAs and act as sponges, alleviating miRNA-mediated gene repression inhibition (Tay et al., 2014). Well-known examples include circRNA CDR1as, a highly stable circRNA rich in conserved binding sites for miR-7 (up to 60) and a potent sponge for miR-7 target genes (Hansen et al., 2013). Many circRNAs are considerably less abundant than miRNAs and may not always act as sponges (Guo et al., 2014); therefore, they may have additional functions.

### 2.2 CircRNAs interact with proteins, which is another important function

EicRNAs interact with the host U1 snRNP and RNA Pol II to inhibit the transcription of their parent genes, competing for mRNA production and affecting protein translation (Ashwal-Fluss et al., 2014). CircRNAs can also act as decoys or scaffolds for proteins. Circ-amotl1 binds and stabilises c-Myc in the nucleus and upregulates its target genes (Yang et al., 2017a). In the cytoplasm, circPABPN1 competes with PABPN1 mRNA to bind to HuR and acts as a HuR decoy, inhibiting PABPN1 translation (Abdelmohsen et al., 2017). Circ-amotl1 acts as a scaffold that bind pdk1 and AKT1 to form ternary complexes, exerting a cardioprotective effect in DOX-induced cardiomyopathy (Zeng et al., 2017). Notably, proteins such as RBPs also regulate circRNA biogenesis (Okholm et al., 2020). FUS binds to the introns flanking back-splicing junctions during back-splicing, altering circRNA expression in mouse embryonic stem cell-derived motor neurones (Errichelli et al., 2017). The interactions between proteins and circRNAs are also worth studying.

### 2.3 Effects on parental gene expression

Some intron-containing circRNAs are immobilised in the nucleus, while ecRNAs are transported to the cytoplasm (Zhang et al., 2013; Li et al., 2015b). Therefore, circRNAs remaining in the nucleus are likely to be involved in transcriptional regulation. As they interact with U1 snRNA and the PoI II complex, EIciRNAs can enhance the transcription of their parental genes (Li et al., 2015b). ciRNA regulates the transcription of its parent genes by promoting the elongation of Pol II (Zhang et al., 2013).

### 2.4 CircRNAs can encode proteins

The source of exonic RNA in the cytoplasm is the protein-coding exon, which is the basis for the translation of circRNAs into proteins. CircRNAs can be translated, directly encoding functional proteins (Yang et al., 2017b; Legnini et al., 2017; Pamudurti et al., 2017). However, further research is required to elucidate the mechanisms underlying circRNA translation.

### 2.5 Competition with linear RNA

As most circRNAs originate from coding gene exons and use the same splicing site and spliceosome as linear RNA, the hypothesis that circRNAs compete with pre-mRNA splicing has emerged (Salzman et al., 2012; Ashwal-Fluss et al., 2014; Starke et al., 2015). CircMBL and its surrounding introns contain consistent MBL-binding sites that are recognised by specific MBL isoforms, contribute to the cyclisation of circMBL, and compete with mRNA splicing (Ashwal-Fluss et al., 2014). Therefore, the competition between circRNAs and linear RNAs may affect gene expression levels, leading to the occurrence of some diseases.

## 3 Mechanisms of the influence of circRNA on IDD

Current research on the specific mechanisms of circRNAs in the development of IDD has focused on five major aspects: cell proliferation and apoptosis, cellular senescence, ECM metabolism, inflammation, and autophagy in IVD cells (Lin et al., 2021; Li et al., 2022a). Pulposus cells are located at the centre of the disc and produce ECM components, whose proliferation and apoptosis play important roles in the pathogenesis of IDD. With age and the development of IDD, the proportion of cells expressing senescence-associated beta-galactosidase (SA-beta-gal) increases, the telomere length shortens, and telomerase activity decreases, indicating varying degrees of senescence in NP cells (Kim et al., 2009; Jeong et al., 2014). The senescence of NP cells affects their ability to proliferate and secrete ECM, promoting the development of IDD. The ECM maintains the structural and functional integrity of the IVDs. Type II collagens and proteoglycans promote ECM synthesis, whereas MMPs and ADAMTS promote ECM catabolism (Vo et al., 2013; Chen et al., 2020a). Imbalances in the proteins related to ECM metabolism affect ECM synthesis and have a direct and profound effect on IDD progression.

Many inflammatory cells and related factors, such as IL-1, TNF-α, and IL-6, have also been found in degenerated IVDs (Lyu et al., 2021). These inflammatory factors affect the development of IDD by activating inflammation-related pathways such as NF-κB, inducing an increase in matrix metalloproteinase levels, and promoting the expression of inflammatory mediators and chemokines, thereby leading to a cascade reaction and exacerbating tissue inflammatory damage (Zhang et al., 2021b). Despite the significance of these mechanisms in IDD, we cannot ignore the role of autophagy in the pathogenesis of IDD (Choi et al., 2016; Xu et al., 2020). Autophagy protects NP cells by scavenging oxidatively damaged mitochondria to prevent cell damage and maintain the energy supply (Ashrafi and Schwarz, 2013; Wang et al., 2018b). Moderately limited autophagy can protect the IVD from degeneration, whereas excessive autophagy or dysfunction caused by various conditions can accelerate the onset of IDD; however, its role in IDD requires further in-depth study (Chen et al., 2017b; Zhang et al., 2020b).

The preceding discussion outlined several contemporary mechanistic approaches concerning the role of circRNAs in IDD. However, a single circRNA can influence IDD through more than one mechanistic pathway. Many circRNAs affect the IDD process via two or more of the aforementioned mechanisms. Based on these findings, we propose the classification of circRNAs into two distinct categories: protective and detrimental circRNAs. This classification depends on whether the circRNAs serve as protective factors for the IVD. Protective circRNAs protect the component structure of the IVD and actively inhibit NP cell senescence and apoptosis, reducing ECM degradation factor production, promoting ECM component expression, inhibiting inflammatory cytokine expression, and regulating the autophagy of IVD cells. In contrast, dermal circRNAs have the opposite effects. Analysing circRNAs from this perspective not only provides a more succinct classification but also supports the practical clinical application of circRNAs in the future. This perspective will help prospective researchers select specific genes for more comprehensive investigations.

**FIGURE 3:**
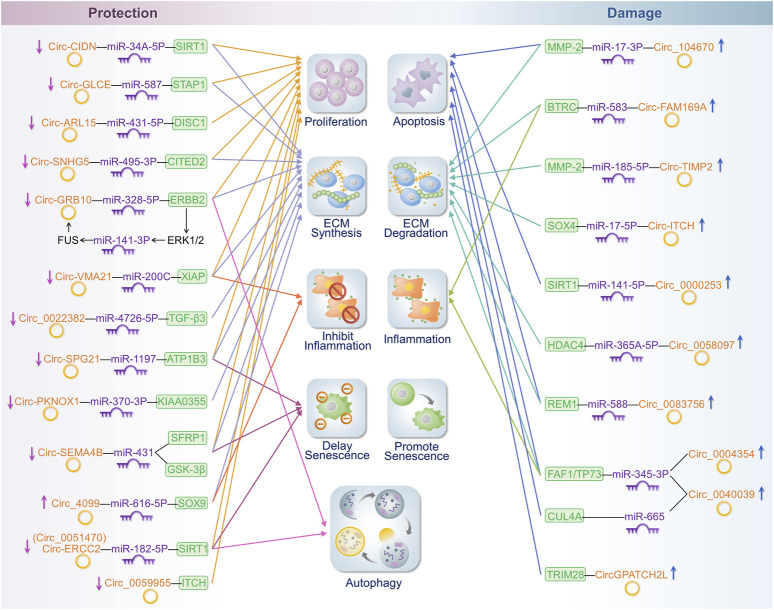
Mechanisms of influence of cyclic RNA in IDD. On the left side of the figure are the circRNAs that protect the intervertebral disc. On the right side of the figure are the circRNAs that play a detrimental role in the intervertebral disc. These circRNAs regulate disease progression in IDD by affecting cell proliferation or the apoptosis, synthesis, or degradation of the ECM; promotion or inhibition of inflammatory responses; and the extent of senescence and autophagy.

The expression of the following circRNAs is significantly reduced or increased in degenerating disc tissues and cells, thereby inhibiting or promoting disc degeneration through the mechanisms described above. Table 1 and Figure 3 show the roles and specific mechanisms of IDD-related circRNAs in detail. Aberrantly expressed circRNAs may serve as diagnostic or therapeutic targets for the clinical treatment of degenerative disc diseases.

**TABLE 1:** Protective and detrimental circRNAs of intervertebral disc.

CircRNA	Dysregulation	Pathway	Function	Type	References
*CIDN*	Downregulated	Circ-*CIDN/mir-34a/SIRT1*	↑NP cell proliferation	Protective gene	Xiang et al. (2020)
			↓ECM degradation		
*GLCE*	Downregulated	Circ-*GLCE/mir-587/STAP1*	↑NP cell proliferation	Protective gene	Chen et al. (2020b)
			↓ECM degradation		
*0072464* (circ*ARL15*)	Downregulated	Circ-*ARL15/mir-431-5p/DISC1*	↑NP cell proliferation	Protective gene	Wang et al. (2021a)
*SNHG5*	Downregulated	Circ-*SNHG5/mir-495-3p/CITED2*	↑NP cell proliferation	Protective gene	Zhang et al. (2021c)
			↓ECM degradation		
*GRB10*	Downregulated	Circ-*GRB10/mir-328-5p/ERBB2*	↑NP cell proliferation	Protective gene	Guo et al. (2018), Guo et al. (2020c)
		↓	↓ECM degradation		
		Erk1/2→*mir-141-3p*→FUS→Circ-*GRB10*	↑Appropriate mitophagy		
*VMA21*	Downregulated	Circ- *VMA21/mir-200c/XIAP*	↑NP cell proliferation	Protective gene	Cheng et al. (2018)
			↓ECM degradation		
			↓Inflammation		
*0022382*	Downregulated	Circ-*0022382/mir-4726-5p/TGF-β3*	↓ECM degradation	Protective gene	Hu et al. (2022)
*0035875* (*SPG21*)	Downregulated	Circ- *SPG21/mir-1197/ATP1B3*	↑NP cell proliferation	Protective gene	Huang et al. (2021b)
			↓ECM degradation		
			↓NP cell senescence		
*PKNOX1*	Downregulated	Circ-*PKNOX1/mir-370-3p/KIAA0355*	↓ECM degradation	Protective gene	Huang et al. (2021a)
*SEMA4B*	Downregulated	Circ-*SEMA4B/mir-431/SFRP1* or GSK-3β/Wnt	↑NP cell proliferation	Protective gene	Wang et al. (2018c)
			↓ECM degradation		
			↓Inflammation		
			↓NP cell senescence		
*4099*	Upregulated	Circ-*4099/mir-616-5p/SOX9*	↓ECM degradation	Protective gene	Wang et al. (2018a)
			↓Inflammation		
*0051470 (ERCC2)*	Downregulated	Circ-*ERCC2/mir-182-5p/SIRT1*	↑NP cell proliferation	Protective gene	Xie et al. (2019)
			↓NP cell senescence		
			↑Appropriate mitophagy		
*0059955*	Downregulated	Circ-*0059955/ITCH*	↑NP cell proliferation	Protective gene	Kong et al. (2020)
*104670*	Upregulated	Circ-*104670/mir-17-3p/MMP-2*	↓NP cell proliferation	Detrimental gene	Song et al. (2018)
			↑ECM degradation		
*FAM169A*	Upregulated	Circ-*FAM169A/mir-583/BTRC*	↑ECM degradation	Detrimental gene	Guo et al. (2020b)
			↑Inflammation		
*TIMP2*	Upregulated	circ-*TIMP2/mir-185-5p/MMP-2*	↑ECM degradation	Detrimental gene	Guo et al. (2020d)
*ITCH*	Upregulated	Circ-*ITCH/mir-17-5p/SOX4*	↑ECM degradation	Detrimental gene	Zhang et al. (2021a)
*0000253*	Upregulated	Circ_*0000253/miR-141-5P/SIRT1*	↓NP cell proliferation	Detrimental gene	Song et al. (2020)
*0058097*	Upregulated	Circ_*0058097/ miR-365A-5p/HDAC4*	↑ECM degradation	Detrimental gene	Xiao et al. (2020)
*0083756*	Upregulated	Circ_*0083756/mir-558/TREM1*	↓NP cell proliferation	Detrimental gene	Du et al. (2022)
			↑ECM degradation		
*0004354*, *0040039*	Upregulated	Circ *0004354 or* Circ *0040039/mir-345-3p/FAF1* or *TP73*	↓NP cell proliferation	Detrimental gene	Li et al. (2022b)
			↑ECM degradation		
			↑Inflammation		
*0040039*	Upregulated	Circ*0040039*/*mir-665/CUL4A*	↓NP cell proliferation	Detrimental gene	Jia et al. (2023)
*GPATCH2L*	Upregulated	CircG*PATCH2L*/*TRIM28*	↓NP cell proliferation	Detrimental gene	Chen et al. (2023)

## 4 Protective circRNAs of IVD

1) The IVD plays an important role as a load-absorbing and cushioning unit in the spine, which endures mechanical stress daily. Excessive mechanical load can lead to apoptosis and ECM degradation in NP cells, promoting IDD (Adams and Roughley, 2006; Yan et al., 2017; Li et al., 2018). Non-coding RNAs influence the IDD process by acting on CEP, which is a new topic for current research (Zhao et al., 2023). Xiang et al. (Xiang et al., 2020) first verified the apoptosis- and catabolism-promoting effects of compression treatments on human NP cells. They found that circRNA-*CIDN* is significantly downregulated in compression-treated human NP cells and that the overexpression of circRNA-*CIDN* inhibits NP cell apoptosis and ECM degradation. The overexpression of *Circ-CIDN* delays IDD progression in an *in vitro* rat IVD organ culture model. This study demonstrates that circRNA-*CIDN* attenuates compressive load damage in human NP cells via the *miR-34a-5p-SIRT1* axis. This study provides the first evidence that circRNAs in IDD can affect human NP cells in compression load-induced injury, providing a new perspective on compression-induced disc degeneration and establishing a library of differentially expressed circRNAs that will facilitate further studies.
2) Chen et al. showed that Circ-*GLCE* is stably present in the cytoplasm of myeloid cells and is significantly downregulated in IDD, as evidenced by microarray and further analysis. The knockdown of Circ-*GLCE* leads to NP apoptosis and the degradation of the NP ECM. In contrast, the overexpression of Circ-*GLCE* partially reverses the apoptosis induced via IL-1β treatment of NP cells. In a mouse model of IDD, the overexpression of Circ-*GLCE* reduces NP cell apoptosis and ECM degradation and delays the progression of IDD *in vivo* via AAV circ-*GLCE* treatment. These results indicate that Circ-*GLCE* attenuates IDD by targeting the *miR-587-STAP1* axis to inhibit apoptosis and ECM degradation in NP cells. This represents a promising therapeutic target for the treatment of IDD.
3) Wang et al. (Wang et al., 2021a) used gene microarray technology and found that circ*ARL15* is the most significantly differentially expressed in the ceRNA network and is downregulated in IDD. Functionally, circ*ARL15* overexpression inhibits apoptosis, and *miR-431-5p* mimics counteract this effect. The direct binding of DISC1 to miR-431-5p was verified via luciferase reporter, co-transfection, and EGFP reporter assays. In a rat model of IDD established by acupuncture, infusion of the circ*ARL15* overexpression plasmid reduced the loss of NP tissue and structural degeneration of the IVD, validating its function *in vivo*. These findings suggest that circARL15 is involved in IDD through the targeting of the *miR-431-5p/DISC1* signalling pathway and regulating NP cell proliferation and apoptosis.
4) CEP is a component of the IVD consisting of hyaline cartilage and extracellular mesenchyme and is responsible for the main blood supply to the disc (Chen et al., 2017b). The disruption of CEP can lead to nutrient deprivation in the ECM and contribute to the induction and development of IDD (Grunhagen et al., 2006). Zhang et al. (Zhang et al., 2021c) investigated the function of circ-*SNHG5* in cartilage endplates in IDD. Initially, a diminished expression of circ-*SNHG5* was observed in degenerative CEP tissues. Through rigorous iterations of knockdown and rescue experiments, we established that circ-*SNHG5* serves as a sponge, inhibiting *miR-495-3p.* Subsequent experiments involving the co-transfection of HEK293T cells with luciferase reporter plasmids and *miR-495-3p* mimics revealed a direct interaction between *miR-495-3p* and *CITED2*, with the former acting as an upstream suppressor. The overexpression of *CITED2* decreased MMP13 levels and increased the expression of collagen II and aggrecan, indicating a healthy ECM. Thus, this study revealed a novel circ-*SNHG5/miR-495-3p/CITED2* axis in IDD, offering promising avenues for future targeted therapies aimed at halting disease progression. Although NP cells have garnered significant attention in IDD research, the relatively unexplored chondrocytes of the CEP may provide valuable insights for future IDD studies. Notably, this study was confined to *in vitro* studies of chondrocytes in the CEP and did not include *in vivo* animal studies. Further investigations are warranted to examine the expression profiles and potential functions of circRNAs in the CEP.
5) Guo et al. (Guo et al., 2018) found that circ*-GRB10* is significantly downregulated in IDD samples; however, it controls the expression of *ERBB2* by sponging *miR-328-5p. ERBB2* induces apoptosis and autophagy by forming a complex with Beclin 1. However, as the reasons behind the downregulation of circ*-GRB10* have not been identified, the authors have conducted a more in-depth study. In further experiments, Guo et al. (Guo et al., 2020c) not only examined the ERBB2/ERK pathway but also investigated the effect of circ-*GRB10* on ECM anabolism. By establishing a rat model of IDD via acupuncture, they found that the overexpression of circ-*GRB10* significantly elevates *ERBB2* levels in NP cells and effectively inhibits the process of IDD in rats. They also elaborated on the upstream mechanisms.

FUS is an RNA-binding protein that regulates the biosynthesis of circ-*GRB10*-forming exons in NP cells by binding to recognition sites (Errichelli et al., 2017). FUS expression is inhibited by targeting *MiR-141-3p*. Finally, *miR-141-3p* is regulated by ERBB2 through the phosphorylation of Erk1/2, as demonstrated via knockdown studies, immunoblotting, and quantitative RT-PCR experiments. Through a series of studies, the authors established a circular signalling pathway formed by circ*-GBR10-miR-328-5p* phosphorylation of the *ERBB2-Erk1/2-miR-141-3p*-Fus loop. Notably, circ-*GRB10* has multiple RBP-binding sites, and the knockdown of FUS does not completely inhibit circ-*GRB10* synthesis. This suggests the existence of regulatory pathways beyond FUS involving circ-*GRB10*, and further studies are needed to verify this hypothesis.

These studies highlight the role and pathway of circ-*GRB10* in IDD; however, research on its clinical aspects is lacking. Wei et al. (Wei et al., 2023) explored the potential of circ-*GRB10* as a biomarker in clinical applications. By comparing the IDD group with the other groups, including sacroiliac joint pain, lumbar disc herniation, piriformis syndrome, entrapment neuropathy, and healthy controls, it was found that plasma circ-GRB10 levels were significantly downregulated. Moreover, after standard treatment in each group, the plasma expression of circ-GRB10 was elevated only in patients with IDD. These results suggest that circ-*GRB10* is specific to lumbar degenerative disc disease. Measuring plasma *GRB10* levels before and after treatment not only improves the diagnostic accuracy of IDD but also suggests its therapeutic efficacy.

6) Cheng et al. (Cheng et al., 2018) found that IDD samples contained considerably lower amounts of circ-*VMA21* than control tissues. Circ-*VMA21*can directly bind to miR-200c in NP cells. Elevated *miR-200c* levels reduce the expression of X-linked inhibitor of apoptosis protein (XIAP), an inhibitor of the apoptotic family of proteins. It binds to cysteoaspartic enzymes that mediate apoptotic cell death, including caspases 3, 7, and 9, and regulates cysteoaspartic enzyme activation, inhibiting apoptosis (Jost and Vucic, 2020). It also regulates several signalling pathways that regulate cell necrosis, autophagy, and differentiation (Hanifeh and Ataei, 2022). In terms of function, the absence of XIAP significantly counteracts the effect of *miR-200c* antagonists on TNF-α- and IL-1β-treated NP cells, inducing apoptosis, exacerbating catabolic responses, and reducing the expression of ECM components. Furthermore, the injection of human adenovirus circ-*VMA21* into punctured IVDs attenuates degenerative changes in the NP and reduces disc degeneration in a rat model of IVDD. These results confirmed that IDD development can be regulated in NP cells through the circ-*VMA21-mir-200C*-*XIAP* axis.
7) Hu et al. (Hu et al., 2022) used human endplate chondrocytes to study IDD because chondrocytes in the endplate can degenerate owing to periodic mechanical tension, and endplate degeneration can lead to IVDD (Holm et al., 2004; Adams and Roughley, 2006; Xiao et al., 2020). The results showed that intermittent cyclic mechanical tension leads to endplate chondrocyte degeneration and circRNA_*0022382* downregulation. CircRNA_*0022382* regulates the anabolic and catabolic activities of the ECM of endplate chondrocytes by sponging *miR-4726-5p* and downregulating the expression of the target gene encoding transforming growth factor 3 (*TGF-β3*). *In vivo* models of acupuncture in rats show that IDD is alleviated by the intravertebral disc injection of circ_*0022382.* Mechanistically, the development of IDD generated by intermittent mechanical tension is effectively blocked by the circ_*0022382-miR-4726-5p-TGF-β3* axis by inhibiting the development of cartilage endplate cell degeneration.
8) Huang et al. (Huang et al., 2021b) analysed clinically normal IVD and degenerated disc tissues and found that water loss and cellular senescence are present in degenerated disc tissues. Moreover, the expression of has_circ_*0035875* (circ*SPG21*) decreases with increasing lesion severity. Transfection experiments showed that the knockdown of circ*SPG21* leads to NP cell senescence, inhibits ECM anabolism, and promotes catabolism; however, the overexpression had the opposite effect. To establish a mouse model of IDD, researchers induced disc degeneration using sustained pressure on the mouse IVD. They also found that injecting circ*SPG21* reverses the increase in ECM breakdown in IDD caused by continuous pressure and slows the development of IDD. Additionally, researchers explored the relationship between circ*SPG21* and *miR-1197* and their target gene *ATP1B3* and confirmed that circ*SPG21* reduces inflammatory cytokine-induced senescence, apoptosis, and ECM catabolism in NP cells via the *miR-1197-ATP1B3* axis. *ATP1B3* is involved in establishing an electrochemical gradient across the plasma membrane for Na^+^ and K^+^ ions, controlling osmotic pressure and sustaining neuromuscular excitement (Mahmmoud, 2005; Ogawa et al., 2015). These results suggest that circ*SPG21* may serve as a biomarker and therapeutic target for the diagnosis of IDD. Furthermore, the downregulation of ATP1B3 may regulate the development of IDD by modulating the exchange of Na^+^/K^+^ ions, leading to cellular water loss. The relationship between IDD development and intracellular ion concentrations requires further investigation.
9) Huang et al. (Huang et al., 2021a) found that circ*PKNOX1* expression is the most downregulated. Immunofluorescence assays and RT-qPCR results indicated that circ*PKNOX1* promotes matrix synthesis and inhibits the breakdown of the corresponding protease. Mechanistically, circ*PKNOX1* regulates *KIAA0355 (GARRE1)* through a pathway mediated by miR-370-3p. *KIAA0355*, a dual effector of Rac and Rab, exhibits low GTPase activity and is intricately linked to a range of cellular activities (Serres et al., 2023). *KIAA0355* knockdown markedly decreases the expression levels of genes associated with ECM formation and increases the expression levels of genes related to ECM degradation in NP cells. This sequence of events accelerates extracellular mesenchymal degradation and promotes IDD progression. The mouse animal model confirmed that increased circ*PKNOX1* expression reverses the trend of decreased anabolism and increased catabolism due to the compression of NP tissue, providing an effective direction for treating IVDD.
10) Wang et al. (Wang et al., 2018c) found that the expression of circ*SEMA4B* is significantly lower in IDD specimens with severe degeneration than in those with mild degeneration, according to the Pfirrmann classification. Inhibiting circ*SEMA4B* enhances the inhibition of proliferation, senescence, and the degradation of the ECM in NP cells via IL-1β; however, its overexpression partially reverses its effects. Mechanistically, circ*SEMA4B* inhibits Wnt pathway activation by competing with the Wnt pathway inhibitors, SFRP1 and GSK-3β, for binding to *mir-431*, thereby regulating proliferation, senescence, and ECM degradation in NP cells and tissues. An miRNA can have multiple target genes acting on the cell, while a circRNA can mediate the development of IDD by regulating these genes. Thus, to comprehensively study the function of circRNAs, attention should be paid to the influence of circRNAs and other non-coding RNA networks.
11) Through circRNA gene microarray and qPCR, Wang et al. (Wang et al., 2018a) showed that circ_*4099* is the most upregulated circRNA in degenerated NP tissues, with a >2-fold change compared to that of normal tissues. TNF-α is upregulated via circ_*4099* through GRP78 in a dose- and time-dependent manner. As the MAPK and NF-κB signalling pathways primarily produce TNF-α, transducing cells with pathway inhibitors, such as sh-p65 and sh-IKKβ, significantly reduces the effect of TNF-α on the induction of expression. Gain- and loss-of-function studies have shown that the overexpression of circ_*4099* increases the expression of type II collagen and aggrecan and significantly inhibits the expression of inflammatory factors but does not regulate MMP-3 and ADAMTS-5. Mechanistically, the expression of circ_*4099* is upregulated by TNF-α via the MAPK and NF-κB pathways and competitively binds *miR-616-5p* to reverse the inhibition of *SOX9*, increasing ECM synthesis in NP cells and playing an important role in ECM metabolism.

Until the present study, circ_*4099* was the only circRNA found to be significantly upregulated in degenerating disc tissues and cells and provided protection to the disc. The upregulation of circ_*4099* is influenced by the action of TNF-α on *GRP78*. *GRP78*, a member of the heat shock protection protein 70 superfamily, is predominantly present in the endoplasmic reticulum (ER) (Kang et al., 2016). As one of the most important ER chaperones, *GRP78* plays an important role in many cellular processes, including protein assembly, folding, and translocation and regulates calcium homeostasis (Wang et al., 2009). *GRP78* expression is upregulated under many stress conditions, including hypoxia and glucose deficiency, regulates cell proliferation, and prevents death (Farshbaf et al., 2020). However, the exact mechanism of *GRP78* and whether it regulates more cirRNAs are unclear, which directs us to further examine the mechanism of cirRNA regulation and explore approaches to significantly upregulate protective circRNAs.

12) Xie et al. (Xie et al., 2019) showed that circ*ERCC2* is the most downregulated gene and functions through the *miR-182-5p/SIRT1* axis. Functionally, the overexpression of circ*ERCC2* significantly reduces apoptosis and increases the phagocytosis of TBHP-treated NP cells (Xia et al., 2015; Guo et al., 2017). revealed that *SIRT1* acts on phagocytosis and apoptosis via the SIRT1-parkin-mitophagy pathway and plays a therapeutic role in IVDD. *SIRT1-*si reduces apoptosis in circ*ERCC2*-inhibited neuronal cells and blocks the inhibitory effects of circ*ERCC2* on NPcells senescence. Rat puncture IDD experiments showed that injecting circ*ERCC2* inhibits NP cell apoptosis and reduces histological grading by enhancing the autophagic response. In conclusion, the circ*ERCC2-miR-182-5P-SIRT1* axis can promote the phagocytosis of cells, reducing apoptosis, delaying senescence, and effectively regulating IDD development.
13) Kong et al. (Kong et al., 2020) studied the effects of circRNA expression on the actions of their host genes. Itchy E3 ubiquitin protein ligase (*ITCH*), the host gene for hsa_circ_*0059955*, promotes the ubiquitination and degradation of p73, a homologue of p53 that induces apoptosis and cell cycle arrest. hsa_circ_*0059955* expression is significantly reduced in IVDD tissues. Downregulating hsa_circ_*0059955* inhibits proliferation, induces apoptosis, and induces G0/G1 phase arrest in NP cells. Moreover, overexpressing hsa_circ_*0059955* significantly attenuates or reverses the progression of IDD in rats. This study suggests that circ_*0059955* may affect the transcription and expression of its host gene *ITCH*, leading to apoptosis and cell cycle arrest in NP cells by inhibiting ITCH. However, they could not completely confirm the action of 0059955 on IDD via ITCH because they did not perform experiments to verify the direct binding between hsa_circ_*0059955* and ITCH. Therefore, the mechanism through which circ_*0059955* affects the protein levels of its host genes requires further investigation.

## 5 Detrimental genes in IVD

1) Song et al. (Song et al., 2018) found that circRNA_*104670* is one of the most upregulated circRNAs, with nearly 4.5-fold upregulation in patients with IDD. Using Cytoscape to construct circRNA–miRNA–mRNA networks and microarray data, they predicted the presence of the circRNA_*104670-miR-17-3p-MMP2* axis, which was experimentally verified. CircRNA_*104670* can induce apoptosis, inhibit the growth of NP cells, and accelerate ECM degradation by inhibiting type II collagen expression and promoting *MMP-2* expression. Through the injection suppression of circRNA_*104670* and the dual gene suppression of circRNA_*104670* and *miR-17-3p*, they found that circRNA_*104670* gene-suppressed mice had lower IDD gradings, while the dual gene suppression group exhibited an accelerated IDD process. By analysing and comparing normal and degenerated human NP tissues, the ROC curves showed that circRNA_*104670* and *miR-17-3p* were of good diagnostic significance for IDD, and both correlated with the IDD grading. This suggests that circRNA_*104670* and *miR-17-3p* may serve as biomarkers for IDD diagnostic grades and as therapeutic targets.
2) Wei et al. (Guo et al., 2020b) analysed circRNAs with specificity using a microarray and found that circ-*FAM169A* is significantly upregulated in degenerative NP tissues and positively correlated with IDD classification (*r* = 0.919). Functionally, circ-*FAM169A* overexpression in NP cells increases the expression of MMP-13 and ADAMTS-5 and decreases the production of type II collagen and aggrecan. The authors injected rats with a circ-*FAM169A* inhibitor after developing a needle-pricked rat model of IDD and showed that inhibiting circ-*FAM169A* significantly protected the structure of the IVD and delayed the process of IDD. *In vitro* and *in vivo* experiments showed that circRNA-*FAM169A* is upregulated in IDD-degenerating tissues and targets it by sponging *miR-583. BTRC* is an E3 ubiquitin ligase that forms the SCF complex through its F-box structural domain. It specifically binds to phosphorylated IκBα and mediates its ubiquitination, facilitating the translocation of NF-κB to the nucleus and activating the transcription of related genes (Spencer et al., 1999; Zhang et al., 2018). NF-κB induces the expression of catabolic genes and exacerbates inflammatory factor responses, shifting the equilibrium toward ECM degradation and facilitating the IDD process (Zhang et al., 2021b).
3) Guo et al. (Guo et al., 2020d) showed that the expression levels of circ-*TIMP2* in NP tissues from patients with IDD are significantly increased, and circ-*TIMP2* directly binds to *miR-185-5p* in these tissues. *MiR-185-5p* is significantly downregulated in TNF-α- and IL-1β-treated cells and degenerated NP tissues. Moreover, *MMP2* is targeted by *miR-185-5p* to regulate NP cell functions. As a metalloprotease, *MMP-2* degrades collagen II and the polymers found in the ECM (Rutges et al., 2008). These results suggest that circ-*TIMP2* acts on NP cells by regulating the expression of *miR-185-5p* and *MMP-2*, promoting ECM degradation. In conclusion, *MMP-2* may act as a target gene for both circ_*104670* and circ-*TIMP2* in IVD. Further comprehensive studies are required to determine whether *MMP-2* has additional regulatory mechanisms.
4) Zhang et al. (Zhang et al., 2021a) revealed that NP tissues from patients with IDD show elevated levels of circ-*ITCH* expression compared to normal tissues. Transfection experiments showed that the knockdown of circ-*ITCH* promotes NP cell proliferation and inhibits ECM degradation; however, circ-ITCH overexpression has the opposite effect. The authors then confirmed that circ-*ITCH* targets and regulates *SOX4* (SRY-box transcription factor 4) gene expression by sponging *miR-17-5p.* The CCK-8 assay showed that circ-*ITCH* overexpression inhibits NP cell proliferation and leads to the degradation of ECM components, whereas circ-*ITCH* knockdown reduces NP cell apoptosis. Moreover, *SO4* regulates the progression of IDD by activating the Wnt/β-catenin pathway. The knockdown of circ-*ITCH* inhibits the expression of Wnt1, β-catenin, c-Myc, and Cyclin d1, and the overexpression of *SOX4*, using the signalling activator LiCl, reverses this response. This finding indicates that circ-*ITCH* is involved in ECM degradation in degenerating NP cells via the *miR-17-5p/SOX4/Wnt/β-catenin* axis.
5) Liao et al. (Liao et al., 2019) found that the *in vivo* delivery of bone marrow-derived mesenchymal stem cell exosomes (BMSC-exos) modulates ER stress-induced apoptosis and delays IDD in a rat caudal model. Therefore, the role of exosomes in IDD needs to be comprehensively explored. Song et al. (Song et al., 2020) collected NP cells media and isolated exosomes and observed that degenerative NP cells secrete more exosomes than normal NP cells. Degenerating NP cells secrete exosomes that contain additional apoptosis-degrading factors which can promote apoptosis in NP cells. CircRNA_*0000253* is the most upregulated circRNA in exosomes generated by degenerating NP cells. Mechanistically, circ_*000253* promotes apoptosis and inhibits cell proliferation by competing for the adsorption of *miR-141-5p* to downregulate *SIRT1*, which promotes IDD both *in vitro* and *in vivo*.

In a rat IDD model established by needling the IVD, the injection of exosomes from the IDD group resulted in IDD in rats, and the severity increased with the grading of IDD from the exosome source. In addition, by injecting AAV-2-circRNA_*0000253*-siRNA into rats, it was found that inhibiting circRNA_*0000253* significantly reduces NP cellsapoptosis and protects the disc structure. Exosomes are vesicles that undergo cellular exocytosis and are composed of lipid-bound nanoparticles (Willms et al., 2018). Exosomes can carry nucleic acids, proteins, mRNAs, and non-coding RNAs and are key mediators of intercellular communication (De Jong et al., 2014). Exosomes are likely to play an important role in the cellular therapy for IDD through various cellular exosome sources as an emerging therapeutic modality (DiStefano et al., 2022; Xia et al., 2022). Notably, Song et al. studied the negative effects of exosomes on the IVD. Their results showed that exosomes variably express different levels of circRNAs in NP cells and act at different levels of disc degeneration, which could be a biological marker for potential therapeutic directions for IDD.

6) Biomechanical stimulation is crucial for the growth and function of endplate cartilage. Excessive mechanical load changes the structure of the endplate cartilage and the components of the ECM, disrupting the nutrient supply, leading to IDD (O'Conor et al., 2013; Vasiliadis et al., 2014). Endplate chondrocytes from primary human endplates were cyclically tensioned at 0.5 Hz with a 10% elongation by Xiao et al. (Xiao et al., 2020). They found altered cytoplasmic morphology and significant downregulation of the AGN, COLII, and SOX9 expression levels in the loaded group compared with those in the control group. The expression of circRNA_*0058097* significantly differed and increased with Thompson grading. The stress resilience of endplate chondrocytes was enhanced by inhibiting circRNA_*0058097* expression. These studies suggest that the tension-sensitive circRNA_*0058097* is present in human endplate chondrocytes and promotes morphological changes in endplate chondrocytes through the circRNA_*0058097-miR-365a-5p-HDAC4* axis, leading to an increase in the extracellular interstitial degradation and degeneration of the endplate cartilage.
7. Du et al. (Du et al., 2022) discovered that hsa_circ_*0083756* expression is increased in tissues and cells with NP degeneration. *In vitro* functional studies showed that circ_*0083756* knockdown promotes NP cell proliferation and ECM synthesis and inhibits the release of inflammatory factors, while gene overexpression had the opposite effect. In a rat IDD model established by acupuncture, inhibiting circ_*0083756* gene expression attenuated the loss of NP tissues and the destruction of IVD structure and reduced the protein expression of ECM degradation and proinflammatory factors. Experiments conducted *in vitro* and *in vivo* indicated that hsa_circ_*0083756* is upregulated in degenerative NP tissues and cells and promotes the IVDD process by sponging *miR-558* to promote *TREM1* expression. The Triggering receptor expressed on myeloid cells-1 (*TREM-1*) gene, located on chromosome 6p21, encodes the TREM-1 glycoprotein receptor, which enhances the expression of proinflammatory cytokines and matrix-degrading enzymes (Panagopoulos et al., 2022). It is likely to promote ECM degradation and apoptosis in NP cells through the TLR4/NF-κB signalling pathway, exacerbating the development of IDD (Zhang et al., 2022).
8) In pathological situations, extensive crosstalk occurs, and cell death commonly occurs in mixed forms rather than in isolation (Karki et al., 2021). Li et al. (Li et al., 2022b) proposed a new proinflammatory cell death modality called PAoptosis, which includes apoptosis and pyroptosis, and is more suitable for studying the mechanisms of inflammatory cell death in IVDD. The apoptotic pathway is mediated by the cleavage of caspase3, which can drive Gasdermin E, leading to pyroptosis with the concomitant secretion of IL-1β (Wang et al., 2017). Current research focuses mostly on the control of miRNAs by a single circRNA; however, the influence of multiple circRNAs belonging to the same parental gene on miRNAs has not been investigated. Syntrophin Beta 2 (SNTB2) is a cytoskeletal protein associated with dystrophin and dystrophin-related proteins that are involved in insulin secretion (Ort et al., 2001; Krautbauer et al., 2019). The authors found that circ_*0040039* and circ_*0004354* are derived from different exon positions of the same gene, *SNTB2*, and both are significantly upregulated in IDD. This study revealed a novel mechanism through which circ_*0040039* and circ_*0004354* induce PAoptosis, a mixed inflammatory cell death, by competitively interacting with *miR-345-3p* and influencing the expression of its target genes *FAF1* and *TP73.* In this study, the authors simultaneously studied two circRNAs, serving as a good demonstration for other researchers to study the effects and mechanisms of interactions between two or even multiple circRNAs in IDD pathogenesis.

Jia et al. (Jia et al., 2023) additionally studied the role of circ_*0040039* (called circ*SNTB2*) in regulating lactate dehydrogenase (LDH) development *in vitro* and *in vivo*. The authors verified that circ*SNTB2* is upregulated in LDH tissues through bioinformatic screening and qRT-PCR. The knockdown of circ*SNTB2* reverses the inhibitory effect of TNF-α treatment on the proliferation of NP cells and the promotion of their apoptosis in both the LDH cell and rat models. Finally, circ*SNTB2* regulate*s CUL4A* by mediating *miR-665*, which inhibits NP cell proliferation and induces apoptosis. However, the study of circ_*0040039* has been extended beyond this point. Huang et al. (Huang et al., 2022) showed via bioinformatic analysis that circ_*0040039* may also act as a sponge for hsa-*miR-424-5p* and hsa-*miR-15b-5p* to regulate downstream genes involved in IDD-related developmental processes through the Wnt/β-catenin signalling pathway. However, this preliminary prediction needs to be supported by further experiments.

From the above results, it can be concluded that the miRNAs sponged by circ_*0040039* (circ*SNTB2*) are different from their target genes. This indicates that the same circRNA can bind to two or more miRNAs and act on different target genes by sponging miRNAs, playing different roles in regulating IDD progression. This suggests that we should pay attention to the crosstalk between circRNAs and the network of miRNAs when studying their mechanism of action and pathways to comprehensively analyse the influence of a certain circRNA on IDD.

9) N6-methyladenosine (m6A) modification is key in regulating gene expression and function and is currently a subject of extensive research. Among the various forms of methylation in eukaryotic cells, m6A modification is the most common and is closely associated with diseases such as tumours, obesity, autoimmune diseases, and neurological disorders (Zhang et al., 2020a). M6A modifications regulate the metabolism and function of circRNAs by affecting circRNA biosynthesis and degradation, cytoplasmic export, and translation; however, m6A modifications are also affected by circRNA interactions (Wang et al., 2021b; Lin et al., 2022). In studies on musculoskeletal disorders, m6A modifications have been found to play a crucial role in regulating the proliferation and migration of tumours, the differentiation of osteoblasts and osteoclasts, chondrocyte function, and the senescence and apoptosis of NP cells (Hu et al., 2022). However, studies on the effect of m6A modifications on circRNAs in IDD are limited.

Chen et al. (Chen et al., 2023) innovatively explored the mechanism and role of m6A methylation in IDD to regulate circ*GPATCH2L*. They screened and verified that circ*GPATCH2L* expression is upregulated in IDD, and m6A methylation is significantly reduced. They demonstrated that upregulated circ*GPATCH2L* inhibits the ubiquitination and degradation of p53 by targeting TRIM28, which impairs DNA damage repair and apoptosis in NP cells. Through a series of experiments, including RNA pulldown, RNA immunoprecipitation, and co-immunoprecipitation, they found that circ*GPATCH2L* regulates the function of TRIM28 proteins by binding to amino acids 402–452 of TRIM28 as a molecular bait, directly targeting TRIM28 proteins. A mouse model of IDD confirmed that the knockdown of circ*GPATCH2L* improves the function of NP cells and ECM metabolism, inhibiting the progression of IDD. Finally, they successfully verified that m6A-methylated circ*GPATCH2L* changes the circ*GPATCH2L* expression levels by cleavage *in vivo* via the YTHDF2-RPL10-RNase P/MRP complex. The results confirmed, for the first time, that circRNAs can act as decoys to interact with target proteins in IDD, regulating the function of target proteins. They also found, for the first time, that m6A modifications alter the progression of IDD by regulating circRNA levels, providing a good model for researchers to study the roles and regulatory mechanisms of circRNAs.

## 6 Potential clinical applications of circRNAs

circRNAs have a great potential for use in various clinical diseases. CircRNAs are highly conserved sequences that lack a 5′ cap and 3′ end, which makes them resistant to degradation by ribonucleases such as ribonuclease R and gives them a longer half-life than linear ribonucleic acids (Harland and Misher, 1988). The technology for identifying and analysing disease-related circRNAs has greatly improved. We identified IDD-related circRNAs via microarray analysis of probes targeting the reverse splice site, which was further confirmed via RT-qPCR (Xu et al., 2021). Furthermore, circRNAs are stable in serum exosomes and human cell-free saliva and are readily detectable in the human body (Li et al., 2015a; Bahn et al., 2015). More importantly, circRNA expression is tissue- or cell-specific (Liu et al., 2016; Yang et al., 2017a).

CircRNAs are stable, easy to detect, and highly specific in the human body with significant potential for clinical diagnostic testing and treatment of IDD and are expected to become biomarkers for IDD and therapeutic targets. Kun et al. (Kun-Peng et al., 2018) demonstrated that serum circ*PVT1* is more reliable than the conventional alkaline phosphatase as a diagnostic tool for identifying patients with osteosarcoma. Circ*PVT1* expression is also significantly upregulated in several types of malignancies, including oesophageal carcinoma, colorectal cancer, gastric cancer, and ERα-positive breast cancer (Chen et al., 2017a; Zhong et al., 2019; Chen and Chen, 2021; RezaSoltani et al., 2023; Yi et al., 2023). This close correlation with a spectrum of clinical conditions makes circ*PVT1* a prospective diagnostic and prognostic biomarker of oncological diseases. CircRNAs are abnormally expressed in degenerating disc tissues and cells and are closely associated with the clinical staging of IDD. CircRNAs are promising biological indicators for the early diagnosis of disc degeneration, initial assessment of IDD severity, and determination of prognosis.

circRNAs bind to miRNAs and affect the expression of their target genes. Notably, a preliminary study by Chen et al. (Chen et al., 2023) found that circ*GPATCH2L* directly binds to target proteins and influences the development of IDD, an area that deserves further research. Current research on circRNAs has revealed that they can accelerate disc degeneration and act as protective factors that slow the progression of IDD. Based on these findings, we can effectively treat degenerative disc disease by overexpressing the circRNAs that protect the disc or by silencing circRNAs that can damage and destroy the disc. This treatment strategy is very promising for the clinical management of IDD, as it is less invasive and can effectively reduce the complications and sequelae caused by surgical procedures.

With the increasing need to regulate miRNA activity, reduce therapeutic costs, and improve production efficiency for disease treatment, artificial circRNAs have been designed. Jost et al. (Jost et al., 2018) designed an artificial *in vitro* transcribed-and-ligated circRNA sponge to isolate miRNA-*122* from the hepatitis C virus (HCV) in a manner similar to that of miravirsen. MiRNA-*122* is closely related to HCV and promotes HCV propagation by binding to sites at the 5′ end of the virus genome. The authors designed an artificial circRNA that effectively inhibits viral protein production by sponging miRNA-122 into a full-length HCV cell culture system. *MiR-21* is overexpressed in gastric cancer (GC) and is associated with the proliferation, invasion, and apoptosis of GC cells.

Liu et al. (Liu et al., 2018) synthesised an artificial circRNA containing five *mir-21* bulged binding sites using an enzymatic binding method, which was able to effectively restore luciferase activity. The degradation rate of LRNA21 was 92% after 30 min in 4% FBS, whereas the synthetic circRNA was more stable and was only degraded at FBS concentrations of 7% or higher. Moreover, synthetic circRNAs inhibit the proliferation of GC cells by upregulating the expression of *DAXX*, a key tumour suppressor gene and a direct target of *miR-21*. Pfafenrot et al. (Pfafenrot et al., 2021) first combined the classical antisense-RNA approach with synthetic short circRNAs, which interfered with the expression of the SARS-COV-2 genome and viral proliferation by specifically targeting SARS-COV-2. Shu et al. (Shu et al., 2018) generated endogenous cRNA sponges via an atypical head-to-tail back-splicing mechanism. Moreover, a user-friendly system was developed to express circulating miRNA inhibitors, which inhibits the function of endogenous miRNAs more potently than its linear counterpart. These designed and synthesised artificial circRNAs are functionally efficient and inexpensive to produce, providing a new approach for inhibiting the *in vitro* functions of targeted miRNAs (Breuer and Rossbach, 2020). The development of synthetic circRNAs requires time for refinement. The mass production of artificial circRNAs in a more efficient manner, monitoring their intracellular stability, and controlling their purification and quality need to be further explored.

## 7 Conclusion and future prospects

This review categorises the circRNAs linked to IDD into two distinct groups: disc-protective and detrimental circRNAs. This classification was derived from a comprehensive examination of the pathogenesis of IDD and the fundamental functions of circRNAs. We scrutinised and dissected the most recent research exploring the correlation between circRNAs and IDD. Given that each study investigating the mechanisms of circRNAs in IDD displays both shared features and unique aspects, we conducted a comprehensive review on the functional mechanism of each circRNA involved in IDD to offer critical insights into each research piece.

Furthermore, we propose the potential of circRNAs as diagnostic indicators or therapeutic agents for the treatment in future clinical scenarios. There is a promising field in which engineered circRNAs could be incorporated into cells to modulate the expression of target genes. These advancements could significantly broaden the scope of genetically targeted therapeutics. Our analysis shows that circRNAs in IDD samples are either upregulated or downregulated, playing a crucial role in the development of IDD, primarily by sponging-related miRNAs to regulate the expression levels of target genes. Therefore, circRNAs affect the proliferation, apoptosis, and senescence of IVD cells and influence the balance between the synthesis and degradation of the ECM and the secretion of inflammatory factors. The conceptualisation and production of artificial circRNAs could help drive further clinical applications of circRNAs in treating related diseases.

Current research on circRNAs is an area of intense interest; however, this is still much to be explored and developed in this field. First, research on the mechanisms involving circRNA in IDD has been focused on sponging miRNAs; however, circRNAs have roles beyond this in interacting with proteins, even in encoding proteins, and influencing parental gene expression. However, there is insufficient research to confirm whether these other functions play profound roles in the development of IDD.

Second, many studies have used a single factor to treat disc cells to build an IDD model. The most commonly used method for inducing disc degeneration is by using single factors such as inflammatory factors (e.g., IL-1 and TNF-a), intermittent cyclic tension, or TBHP, applied to the NP or chondrocytes. However, disc degeneration results from a combination of factors such as cyclic tension, compressive stress, trauma, inflammatory factors, and oxidative stress. Most studies on the therapeutic effects of circRNA-mediated disc degeneration are limited to using the overexpression or silencing of circRNAs to observe their therapeutic effects in single-factor models of IDD. Therefore, even if the overexpression or silencing of circRNAs can reverse the influence of one factor on the IVD, further research is needed to determine whether it affects IDD in cells treated with another factor or a combination of factors.

Third, current research on the mechanisms involving circRNA in IDD focuses on the role played by a single circRNA in the IVD. Nonetheless, several aberrantly expressed circRNAs in degenerating disc tissues and cells have been identified, all of which suppress or accelerate disc degeneration. Li et al. (Li et al., 2022b) revealed the possibility of competition and interaction between two or more circRNAs. Therefore, research efforts need to focus not only on specific circRNAs but also on the interplay and primary and secondary effects of multiple circRNAs in IDD. Thus, we can better understand the underlying mechanism of the entire circRNA population in the IVD.

Fourth, there are many *in vitro* studies of IDD; however, there are few *in vivo* models and clinical studies. Owing to ethical, technical, cost considerations, most *in vivo* studies remain at the animal study stage, and there have been no studies on circRNAs in in vivo models. However, the exact roles and therapeutic effects of circRNAs in humans are not well understood. We need to conduct retrospective and prospective multicentre independent cohort studies in patients with IDD to determine the exact effects of circRNAs in humans. We should also identify a convenient, specific, and reliable method for detecting circRNAs in patients with IDD.

In summary, circRNAs have a great potential for application in degenerative disc diseases. However, our current understanding of the roles and mechanisms of circRNAs in IDD is limited. Thus, further research should be conducted to study the role and regulatory mechanism of circRNAs and build suitable models to verify their clinical value.

## Abbreviations

ADAMTS, A disintegrin and metalloproteinase with thrombospondin-like motifs; AF, annulus fibrosus; BHB, Bulge-helix-bulge; BMSC-exos, Bone marrow-derived mesenchymal stem cell exosomes; CEP, Cartilaginous vertebral endplates; ceRNA, Competitive endogenous; CiRNAs, Circular intronic RNAs; DISC, Death-inducing signalling complex; ECM, Extracellular matrix; EcRNAs, Exonic circular RNAs; EIcRNAs, exonic intronic circular RNAs; FBSS, Failed back surgery syndrome; GC, gastric cancer; HCV, Hepatitis C Virus; IDD, Intervertebral disc degeneration; circRNA:circular RNA; IL-1β, Interleukin-1β, IL-6, Interleukin-6; IVD, Intervertebral disc; LBP, Low back pain; MiRNAs, MicroRNAs; MMP, Matrix metalloproteinases; MREs, MiRNA response elements; NP, Nucleus pulposus; pre-mRNAs, precursor mRNAs; RNA, Δψm: mitochondrial membrane potential; SA-beta-gal, Senescence-associated beta-galactosidase; tgf-β3, Transforming growth factor 3; TNF, tumour necrosis factor; TSEN, tRNA splice endonuclease.
